# Isoenzymatic characterization of *Phlebotomus ariasi* and *P. perniciosus* of canine leishmaniasis foci from Eastern Pyrenean regions and comparison with other populations from Europe

**DOI:** 10.1051/parasite/2018005

**Published:** 2018-02-05

**Authors:** Cristina Ballart, Bernard Pesson, Montserrat Gállego

**Affiliations:** 1 Secció de Parasitologia, Departament de Biologia, Sanitat i Medi Ambient, Facultat de Farmàcia i Ciències de l’Alimentació, Universitat de Barcelona, Av. Joan XXIII 27-31, 08028 Barcelona Spain; 2 ISGlobal, Barcelona Centre for International Health Research (CRESIB), Rosselló 134, 4a planta, 08036 Barcelona Spain; 3 Laboratoire de Parasitologie, Faculté de Pharmacie, Université de Strasbourg, 67401 Illkirch cedex France

**Keywords:** *Phlebotomus ariasi*, *P. perniciosus*, Leishmaniasis, Isoenzymatic characterization, Eastern Pyrenees, Spain

## Abstract

An entomological survey was carried out in 2007 in two Pyrenean counties of Lleida province (north-eastern Spain), where cases of autochthonous canine leishmaniasis have been recently reported. *Phlebotomus ariasi* and *P. perniciosus*, vectors of *Leishmania infantum* in the Mediterranean area, were captured. The aim of the present study was to compare these phlebotomine populations with others captured in known leishmaniasis foci in Europe. Populations of these species were studied by analysing the polymorphism of seven enzymatic systems (HK, PGI, PGM, MDH, 6PGD, FUM and ACO) and compared with other specimens from endemic regions of France, Italy, Malta, Portugal and Spain captured in other campaigns, and also with previously published results. *Phlebotomus* *ariasi* was more polymorphic than *P. perniciosus*. Only the ACO locus had diagnostic alleles, but some other alleles show high characteristic frequencies for each species. The neighbour-joining trees separated two population groups in both species. On the basis of the isoenzyme study results, sand fly populations of the Pyrenean region in Lleida province are closely related to those of other nearby leishmaniasis endemic regions in France and Spain.

## Abstract (fr)

Une enquête entomologique a été réalisée en 2007 dans deux comtés pyrénéens de la province de Lleida (Nord-Est de l’Espagne), où des cas de leishmaniose canine autochtone ont été signalés récemment. *Phlebotomus ariasi* et *P. perniciosus*, vecteurs de *Leishmania infantum* dans la région méditerranéenne, ont été capturés. Le but de la présente étude était de comparer ces populations de phlébotomes avec d’autres capturées dans des foyers connus de leishmaniose en Europe. Les populations de ces espèces ont été étudiées en analysant le polymorphisme de sept systèmes enzymatiques (HK, PGI, PGM, MDH, 6PGD, FUM et ACO) et comparées avec d’autres spécimens de régions endémiques de France, Italie, Malte, Portugal et Espagne capturés dans d’autres campagnes, et aussi avec des résultats déjà publiés. *Phlebotomus ariasi* était plus polymorphe que *P. perniciosus*. Seul le locus ACO possède des allèles diagnostiques, mais certains allèles présentent des fréquences caractéristiques élevées pour chaque espèce. Les arbres produits par Neighbour-joining ont séparé deux groupes de populations chez les deux espèces. Sur la base des résultats de l’étude des isoenzymes, les populations de phlébotomes de la région pyrénéenne dans la province de Lleida sont étroitement liées à celles des autres régions endémiques de leishmaniose voisines en France et en Espagne.

## Introduction

In recent years, several studies on phlebotomine fauna have been carried out in the Mediterranean area, mainly on the vectors of *Leishmania infantum* in the context of the expansion or emergence of leishmaniasis [4,5,24]. In Spain, two sand fly species are proven vectors of *L. infantum*, *Phlebotomus (Larroussius) ariasi* and *P. (L.) perniciosus* [26,32,35]. *P. (L.) ariasi* is associated with cooler and more humid zones, while *P. perniciosus* is ubiquitous and has been found in a wide variety of climatic zones ranging from semi-arid to sub-humid [2,5,6,17,36]. Nevertheless, few studies have been published on population diversity in the new focus areas, including the Pyrenean region of Lleida province in Spain. In this region, two potential vectors with different environmental requirements have been identified, *P. ariasi* and *P. pernicious* [6]. The first is more abundant in the Catalan Pyrenees, where leishmaniasis was not considered endemic until the recent discovery of cases of autochthonous canine leishmaniasis [4,5]. During an entomological survey in 2007 in two Pyrenean counties of Lleida province (north-eastern Spain), sand flies were captured and kept in liquid nitrogen for isoenzymatic characterization of their populations. The use of isoenzymes as genetic markers is well suited to the analysis of a large number of samples. The technique can be used to identify closely related species as well as those non-identifiable *via* morphological criteria alone [13]. The aim of the present study was to compare these populations with others in the neighbouring leishmaniasis foci in France and other European populations. Some of *P. perniciosus* specimens had been studied for three enzymatic systems beforehand (PGI, PGM, HK). In this work, isoelectrofocusing was used to analyse the polymorphism of seven enzymatic systems previously shown to be polymorphic within species of the subgenus *Larroussius* [7,10,11,29], and a comparison was made with other nearby endemic regions and previously published results [8,10,28,30].

## Material and Methods

### Sand fly collection

Population samples were collected from different Mediterranean countries in distinct periods of time (from 1989 to 2009), specifically in France, Italy, Malta, Portugal and Spain, including those in the two Pyrenean counties of Lleida province. The geographical locations with details of the associated code, year of capture, latitude, longitude and altitude are shown in Table 1. A total of 13 populations of *P. perniciosus* (6 from France, 1 from Italy, 1 from Malta, 4 from Spain and 1 from Portugal), and 21 of *P. ariasi* (7 from France, 13 from Spain and 1 from Portugal) were studied. Sand flies were captured using miniature CDC light traps (Centers for Disease Control) [1] set in different locations near farms, houses and kennels. The traps were set for one night at each site at sunset, and left in operation all night (from 6 p.m. to 6 a.m.). The CDC light traps were recovered and grouped according to sampling site and date of capture. Sand flies were immediately cryopreserved in liquid nitrogen (−196°C) until the analysis [10,29].

**Table 1:** Code, year of capture, geographical coordinates and altitude of the populations studied. AR: *Phlebotomus ariasi*; PN: *P. perniciosus*.

Population			Geographical coordinates				
					
	Code	Year of Capture (July–August)	Latitude	Longitude		Altitude (m.a.s.l.)	Studied species
FRANCE							
**Ariège**							
Aston	F-A-AST	2005	42° 45′ 42″ N	1° 39′ 47″ E		647	AR
Limbrassac	F-A-LIM	2006	43° 01′ 03″ N	1° 50′ 34″ E		394	AR/PN
**Aude**							
Arques	F-AU-ARQ	2006	42° 56′ 37″ N	2° 22′ 08″ E		384	PN
Citou	F-AU-CIT	2008	43° 22′ 28″ N	2° 32′ 29″ E		356	AR
Courtauly	F-AU-COU	2006	43° 02′ 58″ N	2° 02′ 27″ E		441	AR
**Dordogne**							
Vaunac	F-D-VAU	2009	45° 21′ 53″ N	0° 52′ 39″ E		199	PN
**Drôme**							
Les Tourrettes	F-DR-TOU	2004	44° 39′ 00″ N	4° 48′ 06″ E		100	AR/PN
**Gard**							
Roquedur	F-G-ROQ	1998	43° 58′ 00″ N	3° 40′ 00″ E		337	AR
**Indre-et-Loire**							
Cinq-Mars-la-Pile	F-IL-CMP	2002	47° 21′ 00″ N	0° 28′ 00″ E		40	PN
**Puy-de-Dôme**							
Glaine-Montaigut	F-PD-GLA	2001	45° 45′ 15″ N	3° 23′ 15″ E		392	PN
**Pyrénées Orientales**							
Vira	F-PO-VIR	2006	42° 46′ 15″ N	2° 24′ 52″ E		310	AR
						
ITALY							
**Apulia**							
Monte Sant'Angelo	I-A-MSA	1991	41° 42′ 13″ N	15° 58′ 50″ E		645	PN
						
MALTA							
**Gozo**							
Zebbug	M-G-ZEB	1989	36° 04′ 29″ N	14° 14′ 18″ E		133	PN
						
SPAIN							
**Girona**							
Sant Jaume de Llierca	E-G-SJL	2002	42° 12′ 05″ N	2° 36′ 25″ E		200	AR
**Huelva**							
Río Tinto	E-H-RTI	1997	37° 41′ 00″ N	6° 35′ 00″ W		415	AR/PN
**Lleida**							
Alins	E-L-ALI	2007	42° 32′ 52″ N	1° 19′ 03″ E		1064	AR
Ainet de Cardós	E-L-AIN	2007	42° 34′ 60″ N	1° 14′ 08″ E		972	AR
Bastida	E-L-BAS	2007	42° 25′ 33″ N	1° 07′ 43″ E		755	AR/PN
Besan	E-L-BES	2007	42° 32′ 10″ N	1° 16′ 51″ E		953	AR
Burg	E-L-BUR	2007	42° 30′ 13″ N	1° 16′ 31″ E		1154	AR
Cassibrós	E-L-CAS	2007	42° 34′ 30″ N	1° 13′ 48″ E		945	AR
Guardia	E-L-GUA	2007	42° 05′ 42″ N	0° 52′ 41″ E		458	AR/PN
Olp	E-L-OLP	2007	42° 25′ 58″ N	1° 06′ 58″ E		1017	AR
Sarroca de Bellera	E-L-SAR	2007	42° 21′ 38″ N	0° 52′ 52″ E		1031	AR
Senterada	E-L-SEN	2007	42° 19′ 51″ N	0° 56′ 25″ E		750	AR
**Tarragona**							
Torroja del Priorat	E-T-TOR	1997	41° 13′ 00″ N	0° 49′ 00″ E		332	AR/PN
						
PORTUGAL							
**Alto Douro**							
Cheires	P-AD-CHE	1996	41° 16' 00" N	7° 31' 60" W		499	AR
Freixo de Espada à Cinta	P-AD-FRE	1996	41° 05′ 12″ N	6° 54′ 68″ W		275	PN

### Enzyme analysis

Sand flies were removed from liquid nitrogen, the last abdominal segments were separated for morphological identification by the keys of Gállego *et al.* (1992) [16] after clearing in Marc-André solution. The rest of the thorax and abdomen were homogenized in 50 μL of distilled water for the isoenzymatic study of the protein extract [11,29].

Isoelectrofocusing was carried out in ultrathin agarose gels with the ampholytes at pH 4.6–5 and 3–10, according to the protocols previously described [7,10]. We studied eight loci of seven polymorphic enzymes in sand flies of the subgenus *Larroussius*: hexokinase (HK, EC 2.7.1.1), glucose phosphate isomerase (PGI, EC 5.3.1.9), phosphoglucomutase (PGM, EC 5.4.2.2), malate dehydrogenase (MDH, EC 1.1.1.37), 6-phosphogluconate dehydrogenase (6PGD, EC 1.1.1.444), fumarase (FUM, EC 4.2.1.2) and aconitase (ACO, EC 4.2.1.3). The alleles were revealed and numbered according to their pHi [10,11,30].

Allele frequencies, tests for deviation from Hardy-Weinberg equilibrium at each locus in each population, and Nei’s genetic distance were calculated using Biosys-2 [38]. PHYLIP version 3.6a2 [14] was used for neighbour-joining phenetic analysis and to calculate bootstrap by majority-rule consensus tree. GENEPOP [34] was used to test genotypic differentiation and to estimate Fst values between each pair of populations. FSTAT v. 2.9.3 [18] was used to test Fst pairwise significance after Bonferroni correction.

## Results

Allele frequencies at eight polymorphic loci are reported separately for *P. perniciosus* and *P. ariasi* (Supplementary Documents 1 and 2). The two species were found to share common alleles. The most polymorphic loci were HK, PGI, PGM and 6PGD, and *P. ariasi* was found to be more polymorphic than *P. perniciosus*. Most of the populations analysed were in Hardy-Weinberg equilibrium, except two out of 13 populations at one locus for *P. perniciosus* (MDH-1), and five out of 21 populations of *P. ariasi* at two loci (PGM and 6-PGD).

Nei’s genetic distances and Fst values between pairs of populations of *P. perniciosus* and *P. ariasi* are given in Tables 2 and 3 , respectively.

**Table 2:** Below diagonal: Fst values between each pair of populations were estimated using GENEPOP [34]. Above diagonal: Nei (1972) [27] genetic distance. Values calculated from the isoenzyme data at the seven polymorphic loci for the 13 populations of *P. perniciosus*. Population codes are given in Table 1.

*P. perniciosus*	E-L-GUA	E-L-BAS	P-AD-FRE	E-T-TOR	E-H-RTI	F-DR-TOU	F-IL-CMP	F-D-VAU	F-PD-GLA	F-A-LIM	F-AU-ARQ	M-G-ZEB	I-A-MSA
E-L-GUA	–	0.007	0.020	0.005	0.011	0.016	0.009	0.009	0.011	0.009	0.010	0.065	0.057
E-L-BAS	0.0063	–	0.024	0.008	0.013	0.007	0.011	0.011	0.010	0.012	0.008	0.070	0.060
P-AD-FRE	0.1371	0.1434	–	0.017	0.013	0.030	0.021	0.021	0.024	0.021	0.023	0.072	0.066
E-T-TOR	0.0303	0.0923	0.2133	–	0.020	0.007	0.001	0.001	0.002	0.001	0.002	0.058	0.051
E-H-RTI	0.0335	0.0511	0.0300	0.1423	–	0.032	0.028	0.028	0.030	0.028	0.027	0.085	0.075
F-DR-TOU	0.1052	0.0411	0.2713	0.0937	0.1956	–	0.006	0.006	0.003	0.008	0.003	0.069	0.061
F-IL-CMP	0.2214	0.3045	0.2366	0.0125	0.2408	0.1379	–	0.000	0.001	0.000	0.001	0.057	0.050
F-D-VAU	0.1746	0.2522	0.1946	0.0096	0.2098	0.0948	-0.0096	–	0.001	0.000	0.001	0.057	0.051
F-PD-GLA	0.0793	0.1020	0.1788	0.0236	0.1632	0.0217	0.0544	0.0254	–	0.002	0.001	0.060	0.054
F-A-LIM	0.2108	0.2561	0.2495	0.0129	0.2486	0.1058	0.0008	0.0039	0.0647	–	0.001	0.057	0.050
F-AU-ARQ	0.0916	0.0946	0.1769	0.0154	0.1605	0.0218	0.0338	0.0119	-0.0155	0.0399	–	0.059	0.053
M-G-ZEB	0.3876	0.3637	0.3609	0.4733	0.3937	0.4684	0.4958	0.4134	0.3818	0.5143	0.3889	–	0.001
I-A-MSA	0.3800	0.3512	0.3689	0.4777	0.3736	0.4756	0.4830	0.4126	0.3870	0.5069	0.3906	−0.0088	–

**Table 3:** Below diagonal: Fst values between each pair of populations were estimated using GENEPOP [34]. Above diagonal: Nei (1972) [27] genetic distance. Values calculated from the isoenzyme data at the seven polymorphic loci for the 21 populations of *P. ariasi*. Population codes are given in Table 1.

*P. ariasi*	E-L-SEN	E-L-SAR	E-L-GUA	E-L-BAS	E-L-OLP	E-L-BUR	E-L-AIN	E-L-CAS	E-L-BES	E-L- ALI	E-G- SIL	E-T-TOR	E-H-RTI	P-AD-CHE	F-A-AST	F-PO-VIR	F-A-LIM	F-AU-COU	F-AU-CIT	F-G-ROQ	F-DR-TOU
E-L-SEN	–	0.004	0.004	0.006	0.006	0.010	0.019	0.021	0.004	0.012	0.006	0.018	0.146	0.110	0.012	0.005	0.011	0.007	0.015	0.012	0.013
E-L-SAR	−0.0059	–	0.002	0.003	0.005	0.007	0.016	0.018	0.001	0.010	0.003	0.010	0.135	0.103	0.006	0.006	0.006	0.003	0.023	0.015	0.012
E-L-GUA	0.0006	−0.0192	–	0.008	0.011	0.014	0.026	0.029	0.005	0.017	0.009	0.016	0.159	0.128	0.013	0.010	0.007	0.008	0.020	0.014	0.012
E-L-BAS	0.0053	−0.0170	0.0051	–	0.002	0.002	0.008	0.009	0.002	0.004	0.002	0.007	0.123	0.087	0.002	0.005	0.007	0.002	0.031	0.023	0.022
E-L-OLP	0.0024	−0.0083	0.0160	−0.0145	–	0.002	0.005	0.007	0.003	0.001	0.001	0.009	0.113	0.076	0.003	0.004	0.008	0.002	0.027	0.023	0.024
E-L-BUR	0.0240	0.0030	0.0301	−0.0159	−0.0168	–	0.003	0.004	0.005	0.002	0.002	0.006	0.113	0.072	0.001	0.007	0.009	0.003	0.038	0.031	0.032
E-L-AIN	0.0571	0.0448	0.0769	0.0088	−0.0062	−0.0108	–	0.001	0.014	0.003	0.006	0.011	0.105	0.061	0.006	0.012	0.018	0.008	0.050	0.045	0.047
E-L-CAS	0.0556	0.0492	0.0778	0.0081	−0.0033	−0.0093	−0.0242	–	0.015	0.004	0.008	0.013	0.103	0.060	0.007	0.013	0.021	0.010	0.054	0.047	0.050
E-L-BES	0.0028	−0.0253	−0.0024	−0.0138	−0.0079	−0.0024	0.0278	0.0307	–	0.007	0.002	0.010	0.127	0.095	0.005	0.005	0.006	0.003	0.024	0.016	0.014
E-L-ALI	0.0225	0.0118	0.0357	−0.0053	−0.0174	−0.0150	−0.0125	−0.0082	0.0038	–	0.002	0.008	0.110	0.071	0.004	0.008	0.010	0.004	0.037	0.033	0.035
E-G-SIL	0.0058	−0.0141	0.0116	−0.0147	−0.0195	−0.0137	0.0006	0.0049	−0.0131	−0.0133	–	0.008	0.113	0.078	0.003	0.004	0.008	0.002	0.027	0.021	0.022
E-T-TOR	0.0743	0.0256	0.0439	0.0203	0.0387	0.0186	0.0462	0.0546	0.0294	0.0372	0.0330	–	0.137	0.099	0.002	0.019	0.006	0.008	0.056	0.046	0.039
E-H-RTI	0.4023	0.3655	0.4263	0.3856	0.3607	0.3642	0.3366	0.3832	0.3726	0.3701	0.3395	0.4339	–	0.024	0.119	0.102	0.148	0.116	0.145	0.131	0.139
P-AD-CHE	0.2686	0.2631	0.2967	0.2335	0.2033	0.2081	0.1773	0.1923	0.2316	0.2014	0.1977	0.2849	0.0711	–	0.083	0.074	0.114	0.083	0.123	0.115	0.125
F-A-AST	0.0384	0.0068	0.0283	−0.0060	0.0030	−0.0080	0.0114	0.0162	0.0030	0.0039	−0.0000	0.0051	0.3855	0.2332	–	0.010	0.006	0.003	0.044	0.034	0.031
F-PO-VIR	0.0088	0.0081	0.0343	0.0128	0.0010	0.0184	0.0315	0.0318	0.0133	0.0152	0.0024	0.0895	0.3286	0.1898	0.0397	–	0.016	0.004	0.016	0.012	0.015
F-A-LIM	0.0364	−0.0080	0.0027	0.0028	0.0163	0.0099	0.0465	0.0527	0.0042	0.0213	0.0116	−0.0020	0.4291	0.2726	−0.0006	0.0608	–	0.006	0.036	0.030	0.026
F-AU-COU	0.0105	−0.0128	0.0064	−0.0109	−0.0129	−0.0101	0.0093	0.0144	−0.0067	−0.0047	−0.0122	0.0301	0.3614	0.2134	0.0028	0.0100	0.0061	–	0.025	0.020	0.020
F-AU-CIT	0.0622	0.0846	0.0892	0.1154	0.0905	0.1424	0.1771	0.1775	0.0908	0.1207	0.0892	0.2097	0.3826	0.2812	0.1591	0.0529	0.1489	0.0877	–	0.004	0.012
F-G-ROQ	0.0446	0.0501	0.0594	0.0783	0.0654	0.1048	0.1412	0.1368	0.0563	0.0925	0.0607	0.1664	0.3439	0.2379	0.1177	0.0304	0.1125	0.0588	−0.0000	–	0.004
F-DR-TOU	0.0422	0.0340	0.0394	0.0637	0.0613	0.0915	0.1331	0.1267	0.0369	0.0831	0.0513	0.1327	0.3190	0.2471	0.0975	0.0447	0.0750	0.0503	0.0407	0.0115	–

**Figure 1:**
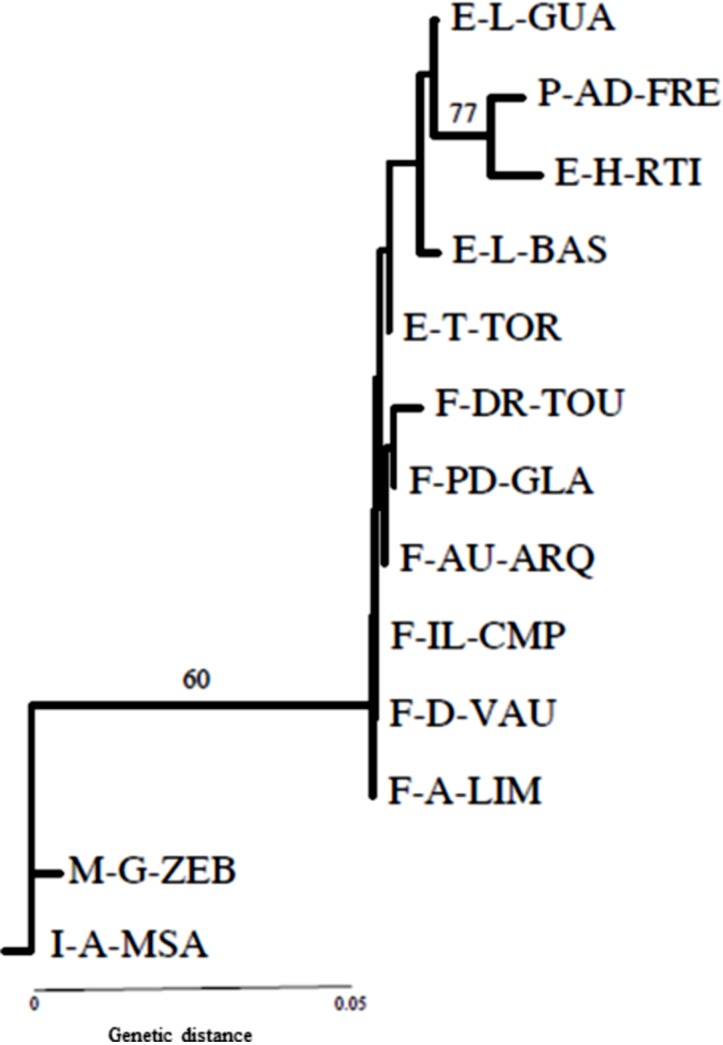
Neighbor-joining phenetic analysis of Nei’s genetic distances among 13 populations of *P. perniciosus*. Neighbor-joining phenetic analysis of Nei’s genetic distances among 13 populations of *P. perniciosus* calculated from allele frequencies at the eight polymorphic isoenzyme loci (GPI: glucosephosphate isomerase; PGM: phosphoglucomutase; HK: hexokinase; FUM: fumarate hydratase; ACO: aconitase; MDH-1: malate dehydrogenase; MDH-2; 6PGD: 6 phosphogluconate dehydrogenase). Bootstrap support values given by 60% majority-rule consensus tree. F-A-LIM: France-Ariège-Limbrassac; F-AU-ARQ: France-Aude-Arques; F-D-VAU: France-Dordogne-Vaunac; F-DR-TOU: France-Drôme- Les Tourrettes; F-IL-CMP: France-Indre-et-Loire-Cinq-Mars-la Pile; F-PD-GLA: France-Puy de Dôme-Glaine-Montaigut; I-A-MSA: Italy-Apulia-Monte Sant’Angelo; M-G-ZEB: Malta-Gozo-Zebbug; E-H-RTI: Spain-Huelva-Río Tinto; E-L-BAS: Spain-Lleida-Bastida; E-L-GUA: Spain-Lleida-Guardia; E-T-TOR: Spain-Tarragona-Torroja; P-AD-FRE: Portugal-Alto Douro-Freixo de Espada á Cinta.

Nei’s genetic distances calculated for the 13 populations of *P. perniciosus* were very low, between 0.000–0.085 and the neighbour-joining tree separated the next groups: one group formed by all the populations of France, Portugal and Spain and other group constituted by Malta and Italy populations, showing a bootstrap value of 60% (Figure 1). The genotypic differentiation for each population pair was highly significant for these two populations. Fst values seemed to confirm the results of the phenetic analysis and point to an intermediary status of *P. perniciosus* populations in France.

**Figure 2:**
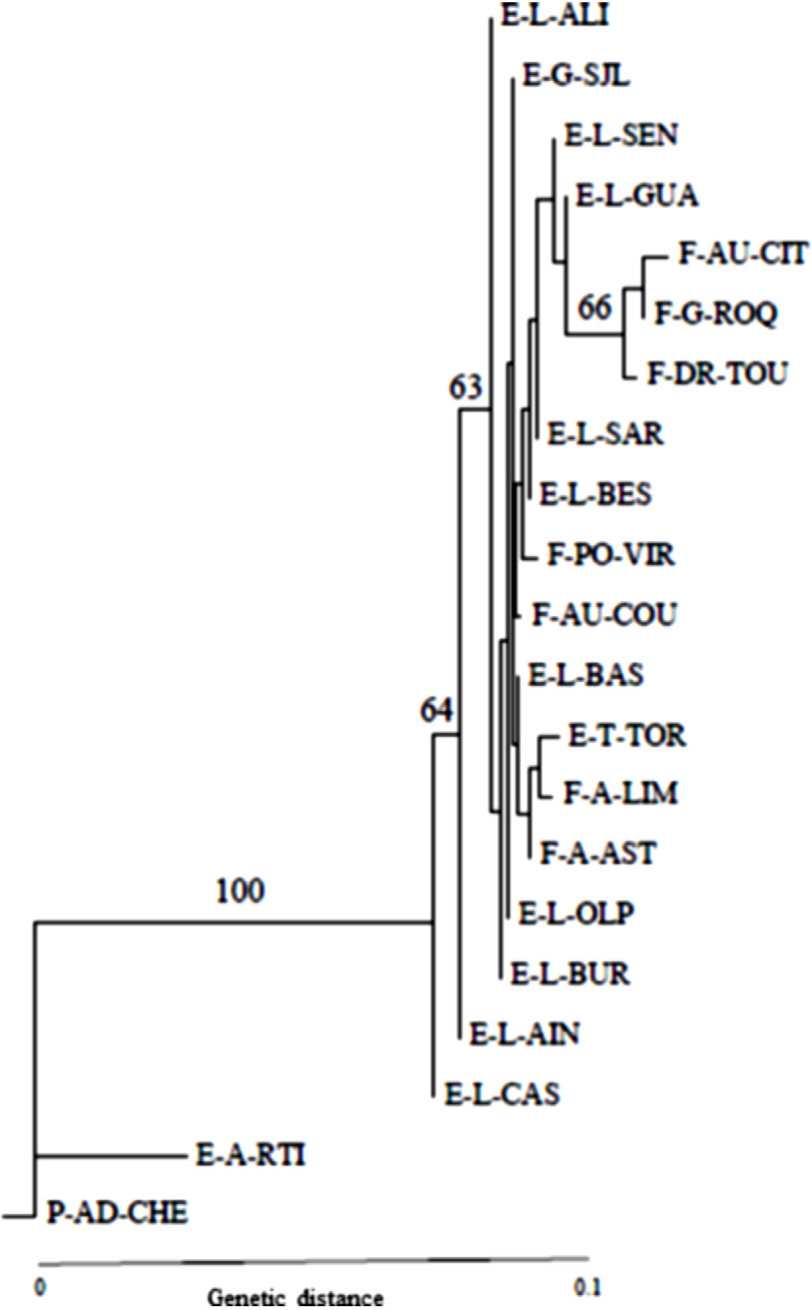
Neighbor-joining phenetic analysis of Nei’s genetic distances among 21 populations of *P. ariasi*. Neighbor-joining phenetic analysis of Nei’s genetic distances among 21 populations of *P. ariasi* calculated from allele frequencies at the eight polymorphic isoenzyme loci (GPI: glucosephosphate isomerase; PGM: phosphoglucomutase; HK: hexokinase; FUM: fumarate hydratase; ACO: aconitase; MDH-1: malate dehydrogenase; MDH-2; 6PGD: 6 phosphogluconate dehydrogenase). Bootstrap support values given by 60% majority-rule consensus tree. F-A-AST: France-Ariège-Aston; F-A-LIM: France-Ariège-Limbrassac; F-AU-CIT: France-Aude-Citou; F-AU-COU: France-Aude-Courtauly; F-DR-TOU: France-Drôme- Les Tourrettes F-G-ROQ: France-Gard-Roquedur; F-PO-VIR : France-Pyrénées Orientales-Vira; E-G-SJL: Spain-Girona-Sant Jaume de Llierca ; E-H-RTI: Spain-Huelva-Río Tinto; E-L-ALI: Spain-Lleida-Alins; E-L-AIN: Spain-Lleida-Ainet de Cardós; E-L-BAS: Spain-Lleida-Bastida; E-L-BES: Spain-Lleida-Besan; E-L-BUR: Spain-Lleida-Burg; E-L-CAS: Spain-Lleida-Cassibrós; E-L-GUA: Spain-Lleida-Guardia; E-L-OLP: Spain-Lleida-Olp; E-L-SAR: Spain-Lleida-Sarroca; E-L-SEN: Spain-Lleida-Senterada; E-T-TOR: Spain-Tarragona-Torroja; P-AD-CHE: Portugal-Alto Douro-Cheires.

Nei’s genetic distances for *P. ariasi* were between 0.001–0.159 and the neighbour-joining tree (Figure 2) separated the western populations (Cheires in Portugal and Río Tinto in Spain), with a bootstrap value of 100%, from the rest of Spain and France, where we found bootstrap values >60%. The genotypic differentiation for each population pair was highly significant for Cheires and Río Tinto populations. As for *P. perniciosus*, Fst values seemed to confirm the results of the phenetic analysis.

In both cases, there was no pairwise significance after Bonferroni correction.

## Discussion

Recently, several studies on phlebotomine fauna have been carried out in Europe regarding the trends for dispersion of leishmaniasis disease [4,5,24]. In the new focus areas, few studies have been published on population diversity. However, studying population diversity more closely in these areas is crucial to detect possible emergence and expansion of the disease. One of these recently discovered foci was the Pyrenean region of Lleida province in Spain, where two potential vectors have been identified, *P. ariasi* and *P. pernicious* [6]. In field work in Lleida, both species were captured at two different times at the same sampling sites, Bastida and Guardia, on cattle and pig farms, respectively, showing its syntopy. In these cases, both species occur sympatrically living in the same geographical area and sharing the possible role of vector species of leishmaniasis [6,35].

In this study, eight gene loci were examined in sand flies from Eastern Pyrenean areas, as in a recent publication [10]. Although three of the enzymes were known to be sufficiently polymorphic for studying populations of *P. perniciosus* (HK, PGI and PGM) [28], there was no previous data available on *P. ariasi*. In our study, all the loci were polymorphic for *P. ariasi* of Lleida, particularly PGI, PGM, ACO and 6-PGD. In the case of the two populations of *P. perniciosus* from Lleida, the most polymorphic were MDH-1 and 6-PGD, whilst HK, FUM, ACO and MDH-2 were monomorphic. The isoenzymatic analyses showed possible diagnostic alleles between the two species only on the ACO locus. ACO 1 was revealed only in *P. perniciosus*. This allele cannot be considered a fixed allele in *P. perniciosus* since one heterozygotic specimen was found in Morocco [10]. Some alleles showed elevated frequencies characteristic for each species: PGI 3, PGM 7, FUM 4 and 6-PGD 2 for *P. ariasi* and PGM 2, FUM 1 and 6-PGD 1 for *P. perniciosus*.

Most of the populations analysed were in Hardy-Weinberg equilibrium, except two out of 13 populations at one locus for *P. perniciosus* (MDH-1), and four out of 21 populations of *P. ariasi* at two loci (PGM and 6-PGD). This disequilibrium could be due to the presence of rare alleles in several individuals with a lack of heterozygotes with the most common allele (MDH-1 for *P. perniciosus* and 6-PGD for *P. ariasi*). In the enzymatic analysis of *P. perniciosus*, only 13 populations were studied because this species is poorly represented in the Pyrenean region (two populations in Lleida and two in France), where high altitudes correlate negatively with its presence [6]. *P. perniciosus* showed low genetic variability. All of the alleles identified in Lleida were found in the other populations, as well as being reported in previous studies [28,29]. The Western populations (Portugal and Huelva) were differentiated by the frequency of allele 2 of the PGI locus, as already observed in other localities of Andalusia and Portugal [7,28,30]. PGM is a polymorphic enzyme that does not structure populations, even on a European scale [28]. In the HK locus, allele 2 was a rare allele of three populations (Italy, Malta and Portugal) and was absent from the other ten populations of Spain and France. However, this allele allowed two groups of *P. perniciosus* to be differentiated, one from North Africa, Malta and Italy, and the other from France and the Iberian Peninsula [28,30]. These results conflict with molecular studies that group populations of France with others of Italy and Malta, suggesting the hypothesis of introgression in France of two lineages from Mediterranean refuges (southern Italy and Spain) during their postglacial dispersal [28]. Our Fst values, like the recent findings with approaches using matrix-assisted laser desorption/ionisation (MALDI-TOF), support an intermediary status of populations in France [23].

The enzymatic analysis of *P. ariasi* involved 21 populations, as this species is well represented in the Pyrenean region (10 populations in Lleida, 1 population in Girona and 4 in France), and correlates positively with increasing altitude [6]. The genetic variability of *P. ariasi* is greater than *P. perniciosus* in the same distribution area [30]. The enzymatic analysis revealed a geographical structuration in two groups: Huelva in south-western Spain and Portugal, supported by allele frequencies of PGI 1, PGM 8, ACO 2 and 6-PGD 8, and showing a bootstrap of 100%. The populations of the Massif Central including the Rhône Valley (North of Aude, Gard and Drôme in France), are supported by allele frequencies of 6-PGD 2 and linked to the remaining populations of the Pyrenean region in Lleida, Spain, South of Aude, Ariège and Pyrénées-Orientales in France, and Tarragona and Girona in Spain, forming a broad group.

No data are available on the vectorial role of *P. perniciosus* in Lleida, but this species has been found naturally infected by *L. infantum* in Tarragona and other regions in Spain, as well as in France, Portugal, Italy and Malta [9,12,19,20,21,25,31,33]. Our results, which include the analysis of a greater number of enzymatic systems, are consistent with those obtained previously with three of these enzymes grouping populations of the Iberian Peninsula and France. In contrast with *P. perniciosus*, more molecular than enzymatic data are available for *P. ariasi* [15,22], and at least four haplogroups have been identified in the European area of distribution. The role of southern Spain as a refuge for *P. ariasi* during the last glaciation has been confirmed [14]. The hypothesis of a complementary refugial area in the northern Pyrenees [22] is supported by our enzymatic analysis, which revealed three rare alleles (PGM 5, HK 8 and 6-PGD 3) only in the Ariège population (Aston) and one (PGM 5) in the Pyrénées-Orientales populations (Vira) and not present in the southern Pyrenean populations in Lleida. As seen before in previous studies, enzymatic and molecular analyses of *P. perniciosus* have yielded differing results. Thus, a combination of different techniques would be recommendable for future studies on the characterization of *P. ariasi* in the Lleida populations. The grouping of the Lleida Pyrenean populations with those analysed from the French Pyrenees (Pyrénées-Orientales and Ariège) and from Girona and Tarragona suggests *P. ariasi* could act as a vector on either side of the mountain range in Spain and France. In fact, the vectorial role of *P. ariasi* in France has been proven [37], while in Lleida only one study has been carried out in a Pyrenean location, where some specimens yielded positive PCR results [3].

## Conclusions

The study of eight loci of seven polymorphic enzymes enabled us to determine that sand fly populations of the Pyrenean region in Lleida province (north-eastern Spain) and of neighbouring Eastern Pyrenees areas are closely related to those of nearby leishmaniasis endemic regions in the other European foci studied.

## Supplementary Material

Tab. S1. Allelic frequencies at the eight polymorphic loci characterized in 13 populations of *P. perniciosus*. Population codes are given in Table 1.
Tab. S2. Allelic frequencies at the eight polymorphic loci characterized in 21 populations of *P. ariasi*. Population codes are given in Table 1. The Supplementary Material is available at https://www.parasite-journal.org/10.1051/parasite/2018005/olm.
