# Supplementary material for: Isoenzymatic characterization of Phlebotomus ariasi and P. perniciosus of canine leishmaniasis foci from Eastern Pyrenean regions and comparison with other populations from Europe
Source: Parasite. 2018 Feb 5;25:3. doi: 10.1051/parasite/2018005 (PMC5798223; doi:10.1051/parasite/2018005)
Supplement: Supplementary file 1 — Tab. S1. Allelic frequencies at the eight polymorphic loci characterized in 13 populations of P. perniciosus. Population codes are given in Table 1. Tab. S2. Allelic frequencies at the eight polymorphic loci characterized in 21 populations of P. ariasi. Population codes are given in Table 1. [file parasite-25-3-s1.zip › parasite170091-1-olm/Supplementary Document 1 _Ballart.doc]

Supplementary Document 1. Allelic frequencies at the eight polymorphic loci characterized in 13 populations of *P. Perniciosus*. Population codes are given in Table 1.

| Populations | | | | | | | | | | | | | |
| --- | --- | --- | --- | --- | --- | --- | --- | --- | --- | --- | --- | --- | --- |
| Locus | E-L-GUA | E-L-BAS | P-AD-FRE | E-T-TOR | E-H-RTI | F-DR-TOU | F-IL-CMP | F-D-VAU | F-PD-GLA | F-A-LIM | F-AU-ARQ | M-G-ZEB | I-A-MSA |
|  |  |  |  |  |  |  |  |  |  |  |  |  |  |
| PGI |  |  |  |  |  |  |  |  |  |  |  |  |  |
| N (pHi) | 14 | 10 | 44 | 89 | 22 | 68 | 36 | 18 | 15 | 37 | 17 | 28 | 40 |
| 1 (5.3) | 0.964 | 1.000 | 0.580 | 0.961 | 0.773 | 1.000 | 1.000 | 1.000 | 1.000 | 1.000 | 1.000 | 0.964 | 1.000 |
| 2 (5.64) | 0.000 | 0.000 | 0.295 | 0.022 | 0.205 | 0.000 | 0.000 | 0.000 | 0.000 | 0.000 | 0.000 | 0.018 | 0.000 |
| 3 (5) | 0.036 | 0.000 | 0.000 | 0.017 | 0.023 | 0.000 | 0.000 | 0.000 | 0.000 | 0.000 | 0.000 | 0.000 | 0.000 |
| 4 (6.19) | 0.000 | 0.000 | 0.000 | 0.000 | 0.000 | 0.000 | 0.000 | 0.000 | 0.000 | 0.000 | 0.000 | 0.018 | 0.000 |
| 5(5.09) | 0.000 | 0.000 | 0.068 | 0.000 | 0.000 | 0.000 | 0.000 | 0.000 | 0.000 | 0.000 | 0.000 | 0.000 | 0.000 |
| 6(5.43) | 0.000 | 0.000 | 0.057 | 0.000 | 0.000 | 0.000 | 0.000 | 0.000 | 0.000 | 0.000 | 0.000 | 0.000 | 0.000 |
| *p* | 1.000 | - | 0.062 | 1.000 | 0.271 | - | - | - | - | - | - | 1.000 | - |
|  |  |  |  |  |  |  |  |  |  |  |  |  |  |
| PGM |  |  |  |  |  |  |  |  |  |  |  |  |  |
| N (pHi) | 13 | 10 | 44 | 89 | 22 | 61 | 36 | 24 | 15 | 38 | 17 | 36 | 40 |
| 1 (4.93) | 0.000 | 0.000 | 0.000 | 0.000 | 0.000 | 0.000 | 0.000 | 0.000 | 0.000 | 0.000 | 0.000 | 0.000 | 0.013 |
| 2 (5.24) | 0.923 | 1.000 | 1.000 | 0.972 | 0.977 | 0.975 | 1.000 | 0.979 | 0.933 | 1.000 | 0.971 | 0.931 | 0.975 |
| 3 (5.1) | 0.077 | 0.000 | 0.000 | 0.006 | 0.000 | 0.000 | 0.000 | 0.000 | 0.067 | 0.000 | 0.000 | 0.028 | 0.000 |
| 4 (5.34) | 0.000 | 0.000 | 0.000 | 0.011 | 0.023 | 0.025 | 0.000 | 0.021 | 0.000 | 0.000 | 0.029 | 0.028 | 0.013 |
| 5 (5.47) | 0.000 | 0.000 | 0.000 | 0.011 | 0.000 | 0.000 | 0.000 | 0.000 | 0.000 | 0.000 | 0.000 | 0.000 | 0.000 |
| 7 (5.29) | 0.000 | 0.000 | 0.000 | 0.000 | 0.000 | 0.000 | 0.000 | 0.000 | 0.000 | 0.000 | 0.000 | 0.014 | 0.000 |
| *p* | 1.000 | - | - | 1.000 | 1.000 | 1.000 | - | 1.000 | 1.000 | - | 1.000 | 1.000 | 1.000 |
|  |  |  |  |  |  |  |  |  |  |  |  |  |  |
| HK |  |  |  |  |  |  |  |  |  |  |  |  |  |
| N (pHi) | 14 | 10 | 43 | 86 | 25 | 67 | 36 | 18 | 15 | 37 | 18 | 28 | 38 |
| 1 (5.6) | 1.000 | 1.000 | 0.953 | 0.994 | 1.000 | 1.000 | 1.000 | 1.000 | 1.000 | 1.000 | 1.000 | 0.357 | 0.382 |
| 2 (5.49) | 0.000 | 0.000 | 0.047 | 0.000 | 0.000 | 0.000 | 0.000 | 0.000 | 0.000 | 0.000 | 0.000 | 0.643 | 0.592 |
| 7 (5.22) | 0.000 | 0.000 | 0.000 | 0.000 | 0.000 | 0.000 | 0.000 | 0.000 | 0.000 | 0.000 | 0.000 | 0.000 | 0.026 |
| 8 (5.41) | 0.000 | 0.000 | 0.000 | 0.006 | 0.000 | 0.000 | 0.000 | 0.000 | 0.000 | 0.000 | 0.000 | 0.000 | 0.000 |
| *p* | - | - | 1.000 | 1.000 | - | - | - | - | - | - | - | 0.239 | 0.509 |
|  |  |  |  |  |  |  |  |  |  |  |  |  |  |
| FUM |  |  |  |  |  |  |  |  |  |  |  |  |  |
| N (pHi) | 5 | 8 | 55 | 63 | 41 | 32 | 25 | 22 | 13 | 17 | 18 | 36 | 49 |
| 1 (6.44) | 1.000 | 1.000 | 1.000 | 0.992 | 0.976 | 0.984 | 1.000 | 1.000 | 1.000 | 0.971 | 1.000 | 1.000 | 0.959 |
| 2 (7.04) | 0.000 | 0.000 | 0.000 | 0.008 | 0.000 | 0.016 | 0.000 | 0.000 | 0.000 | 0.000 | 0.000 | 0.000 | 0.010 |
| 3 (5.8) | 0.000 | 0.000 | 0.000 | 0.000 | 0.012 | 0.000 | 0.000 | 0.000 | 0.000 | 0.029 | 0.000 | 0.000 | 0.010 |
| 5 (6.85) | 0.000 | 0.000 | 0.000 | 0.000 | 0.012 | 0.000 | 0.000 | 0.000 | 0.000 | 0.000 | 0.000 | 0.000 | 0.020 |
| *p* | - | - | - | 1.000 | 1.000 | 1.000 | - | - | - | 1.000 | - | - | 1.000 |
|  |  |  |  |  |  |  |  |  |  |  |  |  |  |
| ACO |  |  |  |  |  |  |  |  |  |  |  |  |  |
| N (pHi) | 5 | 7 | 11 | 15 | 6 | 18 | 15 | 12 | 13 | 16 | 12 | 8 | 9 |
| 1 (8.3) | 1.000 | 1.000 | 1.000 | 1.000 | 1.000 | 1.000 | 1.000 | 1.000 | 1.000 | 1.000 | 1.000 | 1.000 | 1.000 |
| *p* | - | - | - | - | - | - | - | - | - | - | - | - | - |
|  |  |  |  |  |  |  |  |  |  |  |  |  |  |
| MDH1 |  |  |  |  |  |  |  |  |  |  |  |  |  |
| N (pHi) | 5 | 8 | 47 | 65 | 25 | 30 | 51 | 22 | 13 | 17 | 18 | 33 | 37 |
| 1 (5.2) | 1.000 | 0.813 | 0.979 | 0.977 | 0.940 | 0.733 | 0.971 | 0.977 | 0.885 | 1.000 | 0.889 | 1.000 | 1.000 |
| 2 (5.52) | 0.000 | 0.000 | 0.021 | 0.015 | 0.020 | 0.050 | 0.010 | 0.000 | 0.038 | 0.000 | 0.000 | 0.000 | 0.000 |
| 3 (4.85) | 0.000 | 0.188 | 0.000 | 0.008 | 0.040 | 0.217 | 0.020 | 0.023 | 0.077 | 0.000 | 0.083 | 0.000 | 0.000 |
| 5 (5.05) | 0.000 | 0.000 | 0.000 | 0.000 | 0.000 | 0.000 | 0.000 | 0.000 | 0.000 | 0.000 | 0.028 | 0.000 | 0.000 |
| *p* | - | 1.000 | 0.011* | 0.023* | 1.000 | 0.383 | 1.000 | 1.000 | 1.000 | - | 1.000 | - | - |
|  |  |  |  |  |  |  |  |  |  |  |  |  |  |
| MDH2 |  |  |  |  |  |  |  |  |  |  |  |  |  |
| N (pHi) | 5 | 8 | 11 | 32 | 18 | 44 | 51 | 22 | 13 | 17 | 18 | 8 | 9 |
| 1 (8.3) | 1.000 | 1.000 | 1.000 | 1.000 | 1.000 | 1.000 | 1.000 | 1.000 | 1.000 | 1.000 | 1.000 | 0.938 | 1.000 |
| 3 (9.8) | 0.000 | 0.000 | 0.000 | 0.000 | 0.000 | 0.000 | 0.000 | 0.000 | 0.000 | 0.000 | 0.000 | 0.063 | 0.000 |
| *p* | - | - | - | - | - | - | - | - | - | - | - | 1.000 | - |
|  |  |  |  |  |  |  |  |  |  |  |  |  |  |
| 6PGD |  |  |  |  |  |  |  |  |  |  |  |  |  |
| N (pHi) | 5 | 8 | 11 | 33 | 6 | 39 | 25 | 25 | 13 | 18 | 20 | 25 | 9 |
| 1 (6) | 0.700 | 0.750 | 0.818 | 0.909 | 0.583 | 0.949 | 1.000 | 1.000 | 1.000 | 1.000 | 0.975 | 0.920 | 0.889 |
| 7 (6.6) | 0.200 | 0.250 | 0.182 | 0.061 | 0.417 | 0.026 | 0.000 | 0.000 | 0.000 | 0.000 | 0.025 | 0.080 | 0.111 |
| 8 (6.45) | 0.100 | 0.000 | 0.000 | 0.030 | 0.000 | 0.026 | 0.000 | 0.000 | 0.000 | 0.000 | 0.000 | 0.000 | 0.000 |
| *p* | 1.000 | 1.000 | 1.000 | 1.000 | 0.152 | 1.000 | - | - | - | - | 1.000 | 0.120 | 1.000 |

N: sample size; pHi: isoelectric point; PGI: glucosephosphate isomerase; PGM: phosphoglucomutase; HK: hexokinase; FUM: fumarate hydratase; ACO: aconitase; MDH: malate dehydrogenase; 6PGD: 6 phosphogluconate dehydrogenase; *p* = probability of X^2^ value occurring by chance, when testing for deviation from Hardy-Weinberg expectations of genotype frequencies.

* Not in Hardy-Weinberg equilibrium.
