# Supplementary material for: Isoenzymatic characterization of Phlebotomus ariasi and P. perniciosus of canine leishmaniasis foci from Eastern Pyrenean regions and comparison with other populations from Europe
Source: Parasite. 2018 Feb 5;25:3. doi: 10.1051/parasite/2018005 (PMC5798223; doi:10.1051/parasite/2018005)
Supplement: Supplementary file 1 — Tab. S1. Allelic frequencies at the eight polymorphic loci characterized in 13 populations of P. perniciosus. Population codes are given in Table 1. Tab. S2. Allelic frequencies at the eight polymorphic loci characterized in 21 populations of P. ariasi. Population codes are given in Table 1. [file parasite-25-3-s1.zip › parasite170091-1-olm/Supplementary Document 2_Ballart.doc]

Supplementary Document 2. Allelic frequencies at the eight polymorphic loci characterized in 21 populations of *P. ariasi*. Population codes are given in Table 1.

|  | Populations | | | | | | | | | | | | | | | | |  |  |  |  |
| --- | --- | --- | --- | --- | --- | --- | --- | --- | --- | --- | --- | --- | --- | --- | --- | --- | --- | --- | --- | --- | --- |
| Locus | E-L-SEN | E-L-SAR | E-L-GUA | E-L-BAS | E-L-OLP | E-L-BUR | E-L-AIN | E-L-CAS | E-L-BES | E-L-ALI | E-G-SJL | E-T-TOR | E-H-RTI | P-AD-CHE | F-A-AST | F-PO-VIR | F-A-LIM | F-AU-COU | F-AU-CIT | F-G-ROQ | F-DR-TOU |
| PGI |  |  |  |  |  |  |  |  |  |  |  |  |  |  |  |  |  |  |  |  |  |
| N (pHi) | 37 | 19 | 33 | 33 | 35 | 34 | 29 | 33 | 38 | 43 | 35 | 117 | 26 | 79 | 83 | 76 | 43 | 41 | 30 | 43 | 88 |
| 1 (5.3) | 0.405 | 0.342 | 0.318 | 0.318 | 0.400 | 0.353 | 0.414 | 0.409 | 0.342 | 0.395 | 0.386 | 0.188 | 0.731 | 0.722 | 0.277 | 0.500 | 0.233 | 0.378 | 0.633 | 0.570 | 0.466 |
| 2 (5.64) | 0.041 | 0.000 | 0.015 | 0.061 | 0.014 | 0.015 | 0.000 | 0.015 | 0.026 | 0.012 | 0.029 | 0.004 | 0.019 | 0.000 | 0.006 | 0.020 | 0.000 | 0.000 | 0.000 | 0.000 | 0.000 |
| 3 (5) | 0.554 | 0.658 | 0.667 | 0.621 | 0.571 | 0.632 | 0.586 | 0.576 | 0.632 | 0.539 | 0.586 | 0.799 | 0.212 | 0.266 | 0.699 | 0.480 | 0.744 | 0.622 | 0.350 | 0.430 | 0.534 |
| 4 (6.19) | 0.000 | 0.000 | 0.000 | 0.000 | 0.000 | 0.000 | 0.000 | 0.000 | 0.000 | 0.000 | 0.000 | 0.000 | 0.038 | 0.006 | 0.000 | 0.000 | 0.000 | 0.000 | 0.000 | 0.000 | 0.000 |
| 5 (5.09) | 0.000 | 0.000 | 0.000 | 0.000 | 0.014 | 0.000 | 0.000 | 0.000 | 0.000 | 0.000 | 0.000 | 0.000 | 0.000 | 0.000 | 0.006 | 0.000 | 0.000 | 0.000 | 0.000 | 0.000 | 0.000 |
| 8 (5.6) | 0.000 | 0.000 | 0.000 | 0.000 | 0.000 | 0.000 | 0.000 | 0.000 | 0.000 | 0.000 | 0.000 | 0.004 | 0.000 | 0.000 | 0.000 | 0.000 | 0.000 | 0.000 | 0.000 | 0.000 | 0.000 |
| 9 (5.8) | 0.000 | 0.000 | 0.000 | 0.000 | 0.000 | 0.000 | 0.000 | 0.000 | 0.000 | 0.000 | 0.000 | 0.004 | 0.000 | 0.006 | 0.012 | 0.000 | 0.023 | 0.000 | 0.017 | 0.000 | 0.000 |
| *p* | 0.510 | 0.116 | 1.000 | 0.135 | 1.000 | 1.000 | 0.455 | 0.484 | 0.508 | 0.544 | 0.498 | 1.000 | 0.631 | 1.000 | 0.440 | 0.173 | 0.103 | 0.324 | 1.000 | 0.756 | 0.674 |
|  |  |  |  |  |  |  |  |  |  |  |  |  |  |  |  |  |  |  |  |  |  |
| PGM |  |  |  |  |  |  |  |  |  |  |  |  |  |  |  |  |  |  |  |  |  |
| N (pHi) | 30 | 17 | 29 | 33 | 34 | 27 | 19 | 33 | 37 | 42 | 31 | 116 | 26 | 97 | 88 | 81 | 48 | 37 | 35 | 51 | 68 |
| 1 (4.93) | 0.000 | 0.000 | 0.000 | 0.000 | 0.000 | 0.000 | 0.000 | 0.000 | 0.000 | 0.000 | 0.000 | 0.000 | 0.019 | 0.005 | 0.000 | 0.000 | 0.000 | 0.000 | 0.000 | 0.000 | 0.000 |
| 2 (5.24) | 0.000 | 0.000 | 0.000 | 0.000 | 0.029 | 0.000 | 0.000 | 0.000 | 0.000 | 0.000 | 0.000 | 0.004 | 0.000 | 0.021 | 0.000 | 0.000 | 0.000 | 0.000 | 0.000 | 0.000 | 0.000 |
| 3 (5.1) | 0.000 | 0.059 | 0.000 | 0.015 | 0.015 | 0.019 | 0.026 | 0.015 | 0.081 | 0.024 | 0.048 | 0.017 | 0.000 | 0.108 | 0.023 | 0.006 | 0.010 | 0.000 | 0.000 | 0.000 | 0.044 |
| 4 (5.34) | 0.000 | 0.029 | 0.000 | 0.030 | 0.015 | 0.019 | 0.000 | 0.000 | 0.041 | 0.036 | 0.048 | 0.004 | 0.000 | 0.000 | 0.028 | 0.025 | 0.010 | 0.000 | 0.000 | 0.039 | 0.037 |
| 5 (5.47) | 0.000 | 0.000 | 0.000 | 0.000 | 0.000 | 0.000 | 0.000 | 0.000 | 0.000 | 0.000 | 0.000 | 0.000 | 0.000 | 0.000 | 0.006 | 0.012 | 0.000 | 0.000 | 0.000 | 0.000 | 0.000 |
| 6 (5.15) | 0.017 | 0.059 | 0.086 | 0.061 | 0.044 | 0.056 | 0.026 | 0.030 | 0.041 | 0.036 | 0.048 | 0.073 | 0.077 | 0.046 | 0.040 | 0.056 | 0.083 | 0.135 | 0.157 | 0.127 | 0.051 |
| 7 (5.29) | 0.938 | 0.853 | 0.914 | 0.894 | 0.897 | 0.907 | 0.947 | 0.955 | 0.838 | 0.905 | 0.855 | 0.849 | 0.212 | 0.485 | 0.864 | 0.858 | 0.854 | 0.851 | 0.843 | 0.775 | 0.699 |
| 8 (5.45) | 0.000 | 0.000 | 0.000 | 0.000 | 0.000 | 0.000 | 0.000 | 0.000 | 0.000 | 0.000 | 0.000 | 0.013 | 0.692 | 0.335 | 0.034 | 0.043 | 0.021 | 0.014 | 0.000 | 0.049 | 0.051 |
| 10 (5.4) | 0.000 | 0.000 | 0.000 | 0.000 | 0.000 | 0.000 | 0.000 | 0.000 | 0.000 | 0.000 | 0.000 | 0.039 | 0.000 | 0.000 | 0.006 | 0.000 | 0.021 | 0.000 | 0.000 | 0.010 | 0.118 |
| *p* | 1.000 | 0.274 | 0.005* | 0.298 | 0.290 | 0.178 | 1.000 | 0.046* | 0.215 | 0.307 | 0.093 | 0.022* | 0.175 | 0.416 | 0.048* | 0.188 | 1.000 | 0.154 | 0.171 | 0.010* | 0.146 |
|  |  |  |  |  |  |  |  |  |  |  |  |  |  |  |  |  |  |  |  |  |  |
| HK |  |  |  |  |  |  |  |  |  |  |  |  |  |  |  |  |  |  |  |  |  |
| N (pHi) | 37 | 19 | 33 | 33 | 35 | 34 | 29 | 34 | 38 | 43 | 35 | 122 | 29 | 79 | 84 | 79 | 43 | 42 | 31 | 34 | 85 |
| 1 (5.6) | 1.000 | 1.000 | 0.985 | 0.985 | 1.000 | 1.000 | 1.000 | 0.985 | 0.987 | 1.000 | 0.986 | 0.996 | 0.983 | 0.994 | 0.976 | 0.994 | 0.988 | 1.000 | 1.000 | 0.985 | 0.947 |
| 2 (5.49) | 0.000 | 0.000 | 0.015 | 0.015 | 0.000 | 0.000 | 0.000 | 0.015 | 0.013 | 0.000 | 0.000 | 0.000 | 0.017 | 0.000 | 0.000 | 0.006 | 0.000 | 0.000 | 0.000 | 0.000 | 0.000 |
| 4 (5.36) | 0.000 | 0.000 | 0.000 | 0.000 | 0.000 | 0.000 | 0.000 | 0.000 | 0.000 | 0.000 | 0.000 | 0.000 | 0.000 | 0.000 | 0.000 | 0.000 | 0.000 | 0.000 | 0.000 | 0.000 | 0.053 |
| 5 (5.58) | 0.000 | 0.000 | 0.000 | 0.000 | 0.000 | 0.000 | 0.000 | 0.000 | 0.000 | 0.000 | 0.014 | 0.004 | 0.000 | 0.006 | 0.018 | 0.000 | 0.012 | 0.000 | 0.000 | 0.015 | 0.000 |
| 8 (5.41) | 0.000 | 0.000 | 0.000 | 0.000 | 0.000 | 0.000 | 0.000 | 0.000 | 0.000 | 0.000 | 0.000 | 0.000 | 0.000 | 0.000 | 0.006 | 0.000 | 0.000 | 0.000 | 0.000 | 0.000 | 0.000 |
| *p* | - | - | 1.000 | 1.000 | - | - | - | 1.000 | 1.000 | - | 1.000 | 1.000 | 1.000 | 1.000 | 1.000 | 1.000 | 1.000 | - | - | 1.000 | 0.200 |
|  |  |  |  |  |  |  |  |  |  |  |  |  |  |  |  |  |  |  |  |  |  |
| FUM |  |  |  |  |  |  |  |  |  |  |  |  |  |  |  |  |  |  |  |  |  |
| N (pHi) | 24 | 19 | 17 | 18 | 16 | 21 | 18 | 18 | 17 | 17 | 16 | 71 | 27 | 50 | 59 | 42 | 7 | 15 | 17 | 31 | 11 |
| 3 (5.8) | 0.021 | 0.000 | 0.000 | 0.000 | 0.000 | 0.000 | 0.000 | 0.000 | 0.000 | 0.000 | 0.000 | 0.000 | 0.000 | 0.010 | 0.000 | 0.000 | 0.000 | 0.000 | 0.000 | 0.000 | 0.000 |
| 4 (6.16) | 0.958 | 1.000 | 1.000 | 1.000 | 1.000 | 1.000 | 1.000 | 1.000 | 1.000 | 1.000 | 1.000 | 1.000 | 1.000 | 0.990 | 1.000 | 1.000 | 0.929 | 1.000 | 1.000 | 1.000 | 1.000 |
| 5 (6.85) | 0.021 | 0.000 | 0.000 | 0.000 | 0.000 | 0.000 | 0.000 | 0.000 | 0.000 | 0.000 | 0.000 | 0.000 | 0.000 | 0.000 | 0.000 | 0.000 | 0.000 | 0.000 | 0.000 | 0.000 | 0.000 |
| 8 (4.85) | 0.000 | 0.000 | 0.000 | 0.000 | 0.000 | 0.000 | 0.000 | 0.000 | 0.000 | 0.000 | 0.000 | 0.000 | 0.000 | 0.000 | 0.000 | 0.000 | 0.071 | 0.000 | 0.000 | 0.000 | 0.000 |
| *p* | 1.000 | - | - | - | - | - | - | - | - | - | - | - | - | 1.000 | - | - | 1.000 | - | - | - | - |
|  |  |  |  |  |  |  |  |  |  |  |  |  |  |  |  |  |  |  |  |  |  |
| ACO |  |  |  |  |  |  |  |  |  |  |  |  |  |  |  |  |  |  |  |  |  |
| N (pHi) | 12 | 19 | 17 | 17 | 16 | 15 | 18 | 14 | 17 | 17 | 16 | 9 | 6 | 11 | 12 | 12 | 7 | 15 | 17 | 16 | 9 |
| 2 (8.15) | 0.000 | 0.000 | 0.000 | 0.000 | 0.000 | 0.033 | 0.000 | 0.000 | 0.000 | 0.000 | 0.000 | 0.000 | 0.083 | 0.273 | 0.000 | 0.000 | 0.000 | 0.000 | 0.000 | 0.000 | 0.000 |
| 3 (7.95) | 1.000 | 1.000 | 1.000 | 1.000 | 1.000 | 0.967 | 1.000 | 1.000 | 1.000 | 1.000 | 1.000 | 1.000 | 0.917 | 0.727 | 1.000 | 1.000 | 1.000 | 1.000 | 1.000 | 1.000 | 1.000 |
| *p* | - | - | - | - | - | 1.000 | - | - | - | - | - | - | 1.000 | 1.000 | - | - | - | - | - | - | - |
|  |  |  |  |  |  |  |  |  |  |  |  |  |  |  |  |  |  |  |  |  |  |
| MDH1 |  |  |  |  |  |  |  |  |  |  |  |  |  |  |  |  |  |  |  |  |  |
| N (pHi) | 24 | 19 | 15 | 18 | 16 | 21 | 18 | 18 | 17 | 17 | 15 | 72 | 18 | 59 | 48 | 41 | 7 | 12 | 16 | 21 | 15 |
| 1 (5.2) | 0.896 | 0.921 | 0.867 | 0.972 | 0.906 | 0.952 | 0.944 | 1.000 | 0.941 | 0.882 | 0.900 | 0.868 | 1.000 | 0.975 | 0.938 | 0.939 | 0.786 | 0.917 | 0.813 | 0.929 | 0.933 |
| 2 (5.52) | 0.042 | 0.000 | 0.000 | 0.000 | 0.000 | 0.000 | 0.000 | 0.000 | 0.000 | 0.000 | 0.000 | 0.069 | 0.000 | 0.025 | 0.031 | 0.049 | 0.000 | 0.000 | 0.000 | 0.000 | 0.033 |
| 3 (4.85) | 0.063 | 0.026 | 0.033 | 0.028 | 0.094 | 0.048 | 0.028 | 0.000 | 0.059 | 0.118 | 0.067 | 0.035 | 0.000 | 0.000 | 0.031 | 0.012 | 0.143 | 0.083 | 0.188 | 0.071 | 0.033 |
| 4 (5.55) | 0.000 | 0.053 | 0.100 | 0.000 | 0.000 | 0.000 | 0.028 | 0.000 | 0.000 | 0.000 | 0.033 | 0.028 | 0.000 | 0000 | 0.000 | 0.000 | 0.071 | 0.000 | 0.000 | 0.000 | 0.000 |
| *p* | 0.199 | 1.000 | 1.000 | 1.000 | 1.000 | 1.000 | 1.000 | - | 1.000 | 0.176 | 1.000 | 0.093 | - | 1.000 | 0.153 | 1.000 | 1.000 | 1.000 | 1.000 | 1.000 | 1.000 |
|  |  |  |  |  |  |  |  |  |  |  |  |  |  |  |  |  |  |  |  |  |  |
| MDH2 |  |  |  |  |  |  |  |  |  |  |  |  |  |  |  |  |  |  |  |  |  |
| N (pHi) | 24 | 19 | 17 | 18 | 16 | 21 | 18 | 18 | 17 | 17 | 16 | 78 | 11 | 40 | 60 | 43 | 7 | 15 | 17 | 17 | 41 |
| 1 (8.3) | 1.000 | 1.000 | 1.000 | 1.000 | 1.000 | 1.000 | 0.972 | 0.972 | 1.000 | 1.000 | 1.000 | 1.000 | 1.000 | 1.000 | 0.992 | 1.000 | 1.000 | 0.967 | 1.000 | 1.000 | 0.988 |
| 2 (8) | 0.000 | 0.000 | 0.000 | 0.000 | 0.000 | 0.000 | 0.028 | 0.000 | 0.000 | 0.000 | 0.000 | 0.000 | 0.000 | 0.000 | 0.008 | 0.000 | 0.000 | 0.033 | 0.000 | 0.000 | 0.012 |
| 3 (9.5) | 0.000 | 0.000 | 0.000 | 0.000 | 0.000 | 0.000 | 0.000 | 0.028 | 0.000 | 0.000 | 0.000 | 0.000 | 0.000 | 0.000 | 0.000 | 0.000 | 0.000 | 0.000 | 0.000 | 0.000 | 0.000 |
| *p* | - | - | - | - | - | - | 1.000 | 1.000 | - | - | - | - | - | - | 1.000 | - | - | 1.000 | - | - | 1.000 |
|  |  |  |  |  |  |  |  |  |  |  |  |  |  |  |  |  |  |  |  |  |  |
| 6PGD |  |  |  |  |  |  |  |  |  |  |  |  |  |  |  |  |  |  |  |  |  |
| N (pHi) | 24 | 19 | 17 | 17 | 16 | 20 | 18 | 15 | 17 | 17 | 16 | 40 | 9 | 45 | 51 | 50 | 13 | 15 | 24 | 23 | 40 |
| 1 (6) | 0.000 | 0.000 | 0.000 | 0.000 | 0.000 | 0.000 | 0.000 | 0.000 | 0.000 | 0.000 | 0.000 | 0.038 | 0.000 | 0.000 | 0.000 | 0.000 | 0.000 | 0.000 | 0.000 | 0.000 | 0.000 |
| 2 (6.3) | 0.750 | 0.711 | 0.794 | 0.618 | 0.563 | 0.525 | 0.417 | 0.400 | 0.676 | 0.471 | 0.563 | 0.500 | 0.278 | 0.189 | 0.529 | 0.650 | 0.654 | 0.600 | 0.896 | 0.913 | 0.925 |
| 3 (5.91) | 0.000 | 0.000 | 0.000 | 0.000 | 0.000 | 0.000 | 0.000 | 0.000 | 0.000 | 0.000 | 0.000 | 0.000 | 0.000 | 0.000 | 0.010 | 0.000 | 0.000 | 0.000 | 0.000 | 0.000 | 0.000 |
| 4 (5.94) | 0.000 | 0.000 | 0.000 | 0.000 | 0.000 | 0.000 | 0.000 | 0.000 | 0.000 | 0.029 | 0.000 | 0.000 | 0.000 | 0.000 | 0.000 | 0.010 | 0.000 | 0.000 | 0.000 | 0.000 | 0.000 |
| 5 (6.9) | 0.021 | 0.026 | 0.029 | 0.000 | 0.031 | 0.000 | 0.000 | 0.000 | 0.029 | 0.000 | 0.031 | 0.000 | 0.000 | 0.011 | 0.010 | 0.010 | 0.000 | 0.000 | 0.000 | 0.000 | 0.000 |
| 7 (6.6) | 0.000 | 0.000 | 0.000 | 0.000 | 0.000 | 0.000 | 0.000 | 0.000 | 0.000 | 0.029 | 0.000 | 0.025 | 0.000 | 0.000 | 0.010 | 0.000 | 0.000 | 0.000 | 0.000 | 0.000 | 0.000 |
| 8 (6.45) | 0.229 | 0.263 | 0.176 | 0.382 | 0.406 | 0.475 | 0.583 | 0.600 | 0.294 | 0.471 | 0.375 | 0.438 | 0.722 | 0.800 | 0.441 | 0.330 | 0.346 | 0.367 | 0.083 | 0.087 | 0.075 |
| 9 (6.65) | 0.000 | 0.000 | 0.000 | 0.000 | 0.000 | 0.000 | 0.000 | 0.000 | 0.000 | 0.000 | 0.031 | 0.000 | 0.000 | 0.000 | 0.000 | 0.000 | 0.000 | 0.033 | 0.021 | 0.000 | 0.000 |
| *p* | 0.113 | 0.125 | 0.537 | 0.139 | 0.056 | 0.198 | 1.000 | 0.006* | 0.260 | 1.000 | 1.000 | 0.062 | 0.059 | 0.340 | 0.048* | 0.231 | 0.578 | 0.600 | 0.199 | 1.000 | 0.183 |

N: sample size; pHi: isoelectric point; PGI: glucosephosphate isomerase; PGM: phosphoglucomutase; HK: hexokinase; FUM: fumarate hydratase; ACO: aconitase; MDH: malate dehydrogenase; 6PGD: 6 phosphogluconate dehydrogenase; *p* = probability of X^2^ value occurring by chance, when testing for deviation from Hardy-Weinberg expectations of genotype frequencies.

* Not in Hardy-Weinberg equilibrium.
